# Methylated DNA/RNA in Body Fluids as Biomarkers for Lung Cancer

**DOI:** 10.1186/s12575-017-0051-8

**Published:** 2017-03-17

**Authors:** Yan Lu, Shulin/SL Li, Shiguo/SG Zhu, Yabin/YB Gong, Jun/J Shi, Ling/ L Xu

**Affiliations:** 1 No.2 oncology department, Yueyang Hospital of Integrated Traditional Chinese and Western Medicine, Shanghai University of Traditional Chinese Medicine, No.110, Ganhe Rd, Shanghai, China; 2 MD Anderson Cancer Center, the university of Texas, 1840 Old Spanish Trail, Houston, TX USA; 3 Department of Immunology and Pathogenic Biology, School of Basic Medical Sciences, Shanghai University of Traditional Chinese Medicine, 1200 Cai Lun Rd, Shanghai, China

**Keywords:** Lung cancer, Liquid biopsy, ctDNA, Circulating RNA, Methylation

## Abstract

DNA/RNA methylation plays an important role in lung cancer initiation and progression. Liquid biopsy makes use of cells, nucleotides and proteins released from tumor cells into body fluids to help with cancer diagnosis and prognosis. Methylation of circulating tumor DNA (ctDNA) has gained increasing attention as biomarkers for lung cancer. Here we briefly introduce the biological basis and detection method of ctDNA methylation, and review various applications of methylated DNA in body fluids in lung cancer screening, diagnosis, prognosis, monitoring and treatment prediction. We also discuss the emerging role of RNA methylation as biomarkers for cancer.

## Background

Lung Cancer is the second most common malignant tumor and the leading cause of cancer deaths worldwide [1]. Smoking tobacco is the primary risk factor for lung cancer [1, 2]. Early detection and surgery offer the best chance for survival. Screening using low-dose computed tomography (LDCT) has been proved to improve early detection and reduce mortality [3]. However, LDCT is far from satisfactory as a screen tool due to its low specificity [4]. And 30% of patients with as early as stage I lung cancer experience relapse after surgery and recommended adjuvant chemotherapy [5], and for advanced and metastatic disease that is inoperable, patients have to receive radiotherapy, chemotherapy, targeted therapy and immunotherapy and experience remission, recurrence and metastasis. Surveillance plan and treatment decisions are conventionally made based on group statistics and not precise or personalized. The overall 5 year survival of lung cancer is only 17.7% [6]. Therefore, effective biomarkers for early detection, diagnosis, prognosis and monitoring of lung cancer are in urgent need.

Lung cancer is characterized by diverse genetic alterations, making the development of reliable and feasible DNA-based biomarkers very challenging. Epigenetic changes, referred to changes in gene regulation that are not attributed to changes in DNA sequence [7], are relatively consistent in carcinogenesis. Epigenetic abnormalities, comprising alterations in DNA/RNA methylation, histone modifications, nucleosome positioning and noncoding RNAs, are considered hallmarks of cancer initiation and progression [8]. Recent advances in the field of lung cancer epigenetics have revealed promising biomarkers, particularly involving ctDNA methylation and an emerging role of RNA methylation.

## DNA Hypermethylation and Hypomethylation in Lung Cancer

### Hypermethylation

DNA methylation occurs at carbon-5 position of cytosine within CpG dinucleotides that scattered in human genome. The vast majority of the genome contains few CpGs, and most of them are methylated in normal cells. In contrast, around 2% of the genome contains high density of CpG in regions named CpG islands (CGIs) [9] that locate in 50–60% of gene promoters and are often unmethylated during normal development and in adult cells [10]. Methylated CGIs is generally a repressive mark of transcription initiation [11] that hinders the binding of activating transcriptional factors to DNA sequences [12, 13] and recruits inhibitory proteins [14, 15]. The cancer genome is globally hypomethylated, except for the dense methylation at CGIs that is associated with the permanent repression of tumor suppressor genes and other cancer-related genes, thus promotes cancer progression [11, 16]. In non-small cell lung cancer (NSCLC), CGI hypermethylation is associated with diagnosis [17, 18], staging [19], cigarette smoking [20], histological subtype [19, 21, 22], molecular subtypes [23–25], progression [26], prognosis [27–30], and used as a potential therapeutic target [31].

### Hypomethylation

DNA hypomethylation (m5C residues replaced by unmethylated C residues) is the initial epigenetic abnormality recognized in human tumors but has been ignored for a long time [32]. DNA methylation in repetitive sequences could be essential to maintain chromosomal integrity. Studies confirm that DNA hypomethylation is the most constant companion to hypermethylation of the genome in cancer [33–35], lung cancer included [36]. DNA hypomethylation in repetitive sequence occurred in early stage of squamous cell lung cancer [33], and individuals with hypomethylation in repetitive element are at a high risk of developing and dying from cancer [34]. Therefore, hypomethylation could be used as a screening, diagnosis and prognosis biomarker.

## Circulating Tumor DNA (ctDNA) Methylation

### Biological Basis of ctDNA

Circulating cell-free DNA (cfDNA) is a mixture of single or double-stranded DNA in circulation released from different tissues including tumor. Since it is difficult to separate ctDNA from cfDNA originated from non-cancer tissues, careful selection of control group and target genes in a clinical trial is critical. As a result of nuclease digestion during the release processes, cfDNA are usually short fragments with generally very low concentration [37, 38]. In cancer patients, level of cfDNA is elevated with ctDNA as a substantial fraction ranging from < 0.05 [39] to 90% [40], depending on tumor volume, localization, vascularization, hepatic and renal clearance [41]. ctDNA mostly results from apoptosis and necrosis of primary and metastatic tumor [40]. Recent studies have also reported other sources of ctDNA, such as circulating tumor cells (CTCs) [42, 43], and exosomes released by tumor cells [44]. The concordant epigenetic alterations between ctDNA and corresponding tumor tissue DNA [45–48] make ctDNA methylation a promising biomarker for cancer diagnosis and prognosis. Other sources of methylated DNA from liquid biopsy have also been reported, such as cell-surface-bound circulating DNA (csbDNA) [49, 50], Buffy coat DNA [51], peripheral lymphocyte DNA [52, 53], peripheral leukocyte DNA [54, 55], sputum [56] and exhaled breath condensate (EBC) [57].

### ctDNA Extraction Method

cfDNA can be isolated from both plasma and serum. Although cfDNA from serum has been reported with higher quantity [58], its separation process is more demanding to prevent DNA released from the lysis of blood cells [37]. Anyway, it is essential to prepare DNA using very fresh serum/plasma. Therefore, it is highly recommended to process blood sample and separate DNA as soon as possible (within 4 h for serum and 8 h for plasma) [38]. The volume of blood sample necessary to obtain sufficient cfDNA depends on the downstream analysis method. Classical DNA purification methods used for tissues are not suitable for ctDNA [59], and lots of extraction kits designed for cfDNA have become available [59–61].

### ctDNA Methylation Detection Method

Detection of ctDNA methylation has evolved from a few candidate genes to thousands of CpG sites, and recently to whole genome methylation analysis. Detection method of ctDNA methylation can be divided into three groups according to basic principles: sodium bisulfite conversion-dependent methods, restriction enzyme-dependent methods and affinity enrichment-dependent methods.

#### Bisulfite Conversion-Dependent Methods

Sodium bisulfite conversion is the most widely used method to distinguish unmethylated cytosines from methylated ones, and can be coupled with various downstream detection technologies, for example, microarrays, next-generation sequencing (NGS), PCR-based assays, pyrosequencing, quantitative methylation-specific polymerase chain reaction (qMSP) and whole-genome shotgun bisulfite sequencing (WGSBS). Sodium bisulfite rapidly deaminates unmethylated cytosines to uracils, whereas methylated cytosines are only slowly converted [62]. However, bisulfite treatment can induce random DNA breaks, resulting in short single-stranded DNA fragments, especially for cfDNA that is sparse and highly fragmented. Several bisulfite conversion kits with improved recovery of cfDNA have become commercially available, mainly through reducing the incubation time of DNA with bisulfite conversion reagent [63, 64]. Bisulfite treatment also induces reduction in sequence complexity, and cannot distinguish 5-methylcytosines from 5-hydroxymethylcitosines [65], both resulting in compromising efficiency.

#### Restriction Enzyme-Dependent Methods

Restriction enzyme-dependent method utilizes methylation-sensitive restriction enzymes (MSREs) that solely cut unmethylated DNA, so that the rate of false-positives due to incomplete DNA digestion can be prevented. MSREs can be coupled with some downstream detection technologies, for example, differential methylation hybridization (DMH), MCA with microarray hybridization (MCAM), HpaII tiny fragment enrichment by ligation-mediated PCR (HELP), combined bisulfite restriction analysis (COBRA) and methylation-specific multiplex ligation-dependent probe amplification (MS-MLPA). The disadvantage of this method is only a particular pattern of CpG sites can be analyzed.

#### Affinity Enrichment-Dependent Methods

Affinity enrichment-dependent methods utilize specific antibodies interacted with methylated cytosine or methyl-binding proteins to enrich methylated DNA, before further examination with whole-genome analysis by array-based hybridization or next generation sequencing as well as gene-specific determination by PCR. Examples include MethylCpG Binding Domain MBD2 proteins (MBD, also termed Methyl Cap), methylated DNA immunoprecipitation (MeDIP) and methylated CpG island recovery assay (MIRA) [66]. Low recovery rate of methylated DNA is the main disadvantage [67–69].

## Smoking and Lung-Cancer-Related DNA Methylation from Liquid Biopsy

Various factors associated with lung cancer have been shown to alter epigenome that is lung-cancer related, for example aging, chronic inflammation and cigarette smoking [70, 71]. Russo AL et al. report hypermethylation at ECAD, p16, MGMT and DAPK from peripheral lymphocytes DNA as smoking specific epigenome alternation [53]. Baglietto L et al. identified 6 CpGs hypomethylation in 5 genes (AHRR, F2RL3, 2q37.1, 6p21.33 and 12q14.1) from peripheral blood related to smoking that may raise lung cancer risk, and 5 of them were lowest for current smokers and increased with time since quitting for former smokers. Methylation at these 6 CpGs can help improving prediction of lung cancer risk [72]. Gao X et al. demonstrated the impact of tobacco smoking on DNA methylation at 8 lung-cancer-related genes (KLF6, STK32A, TERT, MSH5, ACTA2, GATA3, VTI1A and CHRNA5). DNA hypomethylation in 11 loci was linked to current smokers, compared with never smokers and 10 of them showed significant associations with life-time cumulative smoking [73]. Interestingly, a study from Davis A et al. does not support the association between global blood DNA methylation and the risk of lung cancer in non-smoking women [74], and supports the association between smoking, DNA methylation and lung cancer from opposite side. These studies demonstrate the role of smoking in promoting lung cancer through DNA methylation.

## Methylated DNA from Liquid Biopsy as Biomarkers for Lung Cancer

Tumor-specific methylation of ctDNA are promising biomarkers to help screening, diagnosis, prognosis, monitoring and prediction of therapy response. Due to relatively low efficiency of single biomarker, it is more common to use combination of ctDNA methylations to improve sensitivity and specificity. Some researchers explored the potential role of DNA methylation as a target for lung cancer treatment. Methylated DNA can also be acquired from csbDNA [49, 50], buffy coat DNA [51], and blood cell [75] including lymphocyte [52, 53] and leukocyte [54, 55]. DNA methylations from EBC [57] and sputum [56] are also reported to be associated with lung cancer diagnosis and prognosis.

### Screening and Diagnosis

Methylation occurs at early stage of carcinogenesis, and has become an attractive biomarker for cancer screening and early detection, especially for ctDNA methylation with its convenience and non-invasion. Many studies have reported the potential of ctDNA methylations for the screening and diagnosis of lung cancer. Various gene promoter methylations (Table 1) and their combinations (Table 2) were found to be effective in discriminating lung cancer patients from non-cancer controls. Biomarkers mostly investigated include SHOX2 [46, 47, 76, 77], RASSF1A [54, 75, 78–80], RARB2 [50, 78], LINE-1 [49, 51], P16 [54, 57, 81–83], MGMT [53, 79, 81], DAPK [53, 56, 81], APC [47, 79, 84] and DLEC1 [47, 85]. For example, Powrózek T et al. evaluated DCLK1 methylation status in DNA isolated from peripheral blood plasma from 65 lung cancer patients and 95 healthy individuals. DCLK1 promoter methylation was detected in 32 lung cancer patients (49.2%) and 8 healthy individuals (8.4%). The methylation of the region before transcription start site (TSS) and the region after TSS of DCLK1 gene was detected in 28 and 11 patients, respectively. In seven cases (10.8%), the DCLK1 promoter methylation in both regions was reported. The methylation was observed slightly frequent in patients with small cell lung cancer [17]. Weiss G et al. examined 330 plasma specimens in three independent case–control studies, resulting in a panel of SHOX2 and PTGER4 to distinguish lung cancer from control (area under the receiver operating characteristic curve = 91–98%). A validation study with 172 patient samples demonstrated good performance in distinguishing LC patients from subjects without malignancy (area under the curve = 0.88) [77].

**Table 1:** Single DNA methylation from liquid biopsy as Biomarkers for lung cancer diagnosis

DNA methylation	Body fluid	Method	Number of cases	Number of controls	Sensitivity (%)/specificity (%) or main findings	References
SHOX2	plasma	qMSP	222	189	60/90	[76]
	plasma	qMSP	38	31	80.65/78.57	[46]
DCLK1	plasma	qMSP	65	95	49.2/91.6	[17]
SEPT9	plasma	qMSP	75	100	44.3/92.3	[106]
IEAA	blood	HM450K	43	1986	one unit increase in IEAA was associated with 50% higher risk for LC	[107]
RARβ2	plasma	MSP	52	26	63/51	[50]
	csbDNA	MSP	52	26	70/63	[50]
DLEC1	plasma	MSP	78	50	36/98	[85]
CDH1	serum	qMSP	76	30	62/70	[79]
DCC	serum	qMSP	76	30	35.5/100	[79]
CDH13	plasma	MSP	63	36	33/83	[108]
P16	serum	MSP	22	0	14%	[81]
	plasma	F-MSP	35	15	40/100	[82]
	plasma	modified semi-nested MSP	105	0	73%	[83]
	Plasma	F-MSP	30	30	50%	[57]
	EBC	F-MSP	30	30	40%	[57]
DAPK	serum	MSP	22	0	18%	[81]
	serum	NA	50	0	40%	[80]
GSTP1	serum	MSP	22	0	5%	[81]
MGMT	serum	MSP	22	0	18%	[81]
TMS1	serum	NA	50	0	34%	[80]
RASSFS1A	serum	NA	50	0	34%	[80]
	blood cell	NA	NA	NA	positive with LC diagnosis.	[75]
APC	Serum/plasma	MSP	89	50	47%	[84]
LINE-1	csbDNA	MIRA	56	44	AUC0.69	[49]
	Buffy coat DNA	PCR pyrosequencing	34	360	Hypomethylation is associated with 3.2-fold higher risk for LC	[51]
p53	peripheral lymphocyte DNA	HpaII quantitative PCR	100	-	Hypomethylation was associated with a 2-fold increased risk for LC	[52]

*qMSP* quantitative methylation-specific PCR, *F-MSP* fluorescent methylation-specific PCR, *HM450K* HumanMethylation450K BeadChip Assay, *MSP* methylation-specific PCR, *MIRA* methylated CpG island recovery assay, *LC* lung cancer, *SHOX2* short stature homebox 2, *DCLK1* doublecortin like kinase 1, *SEPT9* septin9, *IEAA* intrinsic epigenetic age acceleration, *RARβ2* retinoic acid receptor B2, *DLEC1* Deleted in lung and esophageal cancer 1, *CDH1* cadherin 1, *DCC* DCC netrin 1 receptor, *CDH13* cadherin 13, *DAPK* death-associated protein kinase, *GSTP1* glutathione S-transferase P1, *MGMT* O6 - methylguanine-DNA-methyltransferase, *RASSF1A* ras association domain family 1 isoform A, *APC* adenomatous polyposis coli, *p16* cyclin-dependent kinase inhibitor 2A, *csbDNA* cell-surface-bound circulating DNA, *EBC* exhaled breath condensate, *NA* not available

**Table 2:** Combination of DNA methylation from liquid biopsy as Biomarkers for lung cancer diagnosis

DNA methylation combination	Body fluid	Method	Number of cases	Number of controls	Sensitivity%/specificity% or main findings	References
RASSF1A/RARB2	Plasma/csbDNA	qMSP	60	32	87/75	[78]
SHOX2/PTGER4	plasma	Rt-PCR	117	122	67/90 or 90/73	[77]
RTEL1/PCDHGB6	cfDNA	qMSP-PCR	70	80	62.9/90 (AUC0.755)	[17]
HOXD10/PAX9/PTPRN2/STAG3	serum	MSRE/qPCR	23	23	87.8/90.2	[109]
APC/RASSF1A/CDH13/KLK1/DLEC1	plasma	MSP	110	50	83/70	[47]
APC/AIM1/CDH1/DCC/MGMT/RASSF1A	serum	qMSP	76	30	84/57	[79]
DAPK/PAX5b/PAX5a/Dal1/GATA5/SULF2/CXCL14	sputum	MSP	40	90	75/68	[56]
MGMT/DAPK/PAX5β/Dal-1/PCDH20/Jph3/Kif1a			64	64		
CSF3R/ERCC1	peripheral leukocyte	pyrosequencing	138	138	Predict higher risk for SCLC	[55]
CDH1/p16/MGMT/DAPK	peripheral lymphocyte	MSP	49	22	methylation of CDH1 and DAPK occurs in the early stages LC	[53]
					methylation of p16 and MGMT occurs in later stages LC	
p16/RASSF1A/FHIT/RTL	WBC DNA	SYBR Green-based qMSP and qPCR	200	200	AUC 0.670–0.810	[54]

*qMSP* quantitative methylation-specific PCR, *Rt-PCR* real-time PCR, *MSRE* Methylation-Sensitive Restriction Enzymes, *qPCR* quantitative PCR, *csbDNA* cell-surface-bound circulating DNA, *LC* lung cancer, *SCLC* small cell lung cancer, *RASSF1A* ras association domain family 1 isoform A, *RARβ2* retinoic acid receptor B2, *SHOX2* short stature homebox 2, *PTGER4* prostaglandin E receptor 4, *RTEL1* regulator of telomere elongation helicase 1, *PCDHGB6* protocadherin gamma subfamily B, 6, *HOXD10* homeobox D10, *PAX9* paired box 9, *PTPRN2* protein tyrosine phosphatase receptor type N2, *STAG3* stromal antigen 3, *APC* adenomatous polyposis coli, *DLEC1* Deleted in lung and esophageal cancer 1, *CDH13* cadherin 13, *KLK1* kallikrein 1, *AIM1* absent in melanoma 1, *CDH1* cadherin 1, *DCC* DCC netrin 1 receptor, *MGMT* O6 - methylguanine-DNA-methyltransferase, *DAPK* death-associated protein kinase, *PAX5b* paired box 5b, *PAX5a* paired box 5a, *GATA5* GATA binding protein 5, *SULF2* sulfatase 2, *CXCL14* C-X-C motif chemokine ligand 14, *PCDH20* protocadherin 20, *Jph3* junctophilin 3, *CSF3R* colony stimulating factor 3 receptor, *ERCC1* ERCC excision repair 1, *FHIT* fragile histidine triad, *RTL* relative telomere length, *p16* cyclin-dependent kinase inhibitor 2A

A large proportion of results mentioned above are based on studies comparing advanced lung cancer with healthy control. To avoid bias and improve the screening and early diagnosis efficiency, studies should include specifically early stage LC and non-cancer control.

### Monitoring and Prognosis

DNA methylation can be used to indicate risk of cancer recurrence due to residual disease after surgery/chemotherapy. Due to its short half-life, ctDNA can reflect tumor burden sensitively and allows ‘real-time’ monitoring of tumor dynamics. Persistence of ctDNA in blood after surgery is associated with poor prognosis [39]. In early stage like stage Ib NSCLC, benefit from adjuvant chemotherapy is controversial, and ctDNA methylation might be used as a prognostic biomarker to define patients at high risk of recurrence who may benefit from chemotherapy. In patients with high probability of recurrence after surgery, monitoring with ctDNA methylation can be a good surrogate to image and tumor markers, and improve clinical outcome with early detection of recurrence [45, 86]. Ponomaryova AA et al. investigated the methylation status in plasma of 32 healthy donors and 60 lung cancer patients before and after treatment, and found that chemotherapy and total tumor resection resulted in a significant decrease in the index of methylation for RARB2 and RASSF1A, and methylation of RARB2 detected within follow-up period manifested disease relapse at 9 months [78]. Schmidt B et al. demonstrated better survival in patients with low SHOX2 promotor methylation 1 week after the start of chemotherapy [87]. In advanced and metastatic lung cancer, some biomarkers are associated with disease progression and survival, including BRMS1 [86], SOX17 [45], DCLK1 [17], and SFN (14-3-3 Sigma) promoter methylation [88] (Table 3).

**Table 3:** Methylation of DNA from liquid biopsy as biomarkers for lung cancer prognosis and prediction

DNA methylation	Body fluid	Method	Number of cases	Number of controls	Main findings	References
SHOX2	plasma	qMSP	36	-	negative impact on survival	[87]
RARB2/RASSF1A	plasma	qMSP	26	-	Reduced after neoadjuvant chemotherapy and surgery;	[78]
RARB2	plasma	qMSP	26	-	increased before recurrence	[78]
RASSF1A/APC	plasma	qMSP	316	-	Elevated after chemotherapy; correlated with good response to cisplatin	[89]
DCLK1	plasma	qMSP	65	95	negative impact on survival	[17]
BRMS1	plasma	qMSP	122	24	negative impact on survival	[86]
SOX17	plasma	qMSP	122	24	negative impact on survival	[45]
SFN	serum	qMSP	115	-	positive impact on survival with platinum-based chemotherapy	[88]
CHFR	serum	qMSP	366	-	negative impact on survival with second-line EGFR-TKIs, compared to chemotherapy	[90]
smoCpGs	Whole blood	HM450K	60	1505	predict LC mortality (HR7.82)	[110]
APC/RASSF1A/CDH13/CDKN2A	Plasma	MSP	45	-	negative impact on PFS and OS	[31]

*qMSP* quantitative methylation-specific PCR, *HM450K* HumanMethylation450K BeadChip Assay, *LC* lung cancer, *PFS* progression free survival, *OS* overall survival, *SHOX2* short stature homebox 2, *RARβ2* retinoic acid receptor B2, *RASSF1A* ras association domain family 1 isoform A, *APC* adenomatous polyposis coli, *DCLK1* doublecortin like kinase 1, *BRMS1* breast cancer metastasis suppressor-1, *SOX17* (sex determining region Y)-box 17, *SFN* stratifin, *CHFR* checkpoint with forkhead and ring finger domains, *smoCpGs* smoking-associated CpGs, *caCpGs* Lung cancer-related CpGs, *CDH13* cadherin 13, *CDKN2A* cyclin dependent kinase inhibitor 2A

### Prediction of Therapy Response

ctDNA provides an potential detection of early response to treatment, compared with conventional imaging or protein based biomarkers. Several studies have reported the use of tumor-specific methylation to track patient’s response to therapy (Table 3). For example, Wang H et al. reported an elevated level of APC and RASSF1A promoter methylation in ctDNA within 24 h after cisplatin-based therapy, consistent with chemotherapy induced cell death [89]. The value of methylated ctDNA to predict response to therapy has also been investigated. For example, Salazar F et al. reported that patients with unmethylated CHFR promoter survived longer when receiving EGFR tyrosine kinase inhibitors as second-line treatment, compared to conventional chemotherapy [90].

### Target for Therapy

With the significance of DNA methylation in cancer progression, epigenetic treatment became a potential therapeutic candidate. Effect of epigenetic therapy in lung cancer has been reported. Juergens RA et al. investigated combined epigenetic therapy with azacitidine and entinostat, inhibitors of DNA methylation and histone deacetylation respectively, in patients with recurrent metastatic NSCLC, and with demethylation of a set of 4 epigenetically silenced genes known to be associated with lung cancer in serial blood samples, resulted in objective and durable responses [31]. Further investigations of methylated ctDNA as a treatment target are expected.

## Emerging Role of RNA Methylation

RNA methylation was first described as a form of post-transcriptional modification more than 40 years ago [91, 92]. But the exact mechanism and significance of methylated RNA is just beginning to be appreciated. Among more than a hundred types of nucleotide modifications identified in different RNA molecules [93, 94], m6A modification has attracted most attention owing to its potential to regulate gene expression reversibly. RNA with m6A modification does not activate TLR3 [95, 96], leading to non-recognition of viral components, and may stimulate a pathway involved in cancer development [96–98]. RNA methylation may alter miRNA expression and mediate cancer cell migration [99]. RNA methylation may be involved in cancer stem cells specification and disease progression [100]. The application of circulating RNA methylation in various types of cancer has been reported. For example, Muraoka T et al. proved that serum miR-34b/c methylation can be used for the diagnosis and prognosis of malignant pleural mesothelioma [101]. Drugs that induced RNA demethylation might contribute to patient responses [102, 103]. Lian CG et al. reported another type of modification in RNA, 5-hydroxymethyl cytosine (5hmC), as a signature for melanoma prognosis [104]. Further research on circulating RNA methylation in lung cancer is anticipated.

## Conclusions and Perspectives

Lung cancer liquid biopsy has received increasing attention in recent years with its advantage as a non-invasive detection. Among the huge amount of information obtained from liquid biopsy, epigenetic alterations, especially DNA/RNA methylation, has been widely researched. ctDNA/RNA methylation has been associated with the screening, diagnosis, prognosis, monitoring and treatment prediction of lung cancer. The advances in techniques enable detection of methylation from sparse and fragmented DNA/RNA. For example, it is now feasible to detect DNA/RNA methylation from single CTC [105]. However, the methodology is still in lack of standardization, which hinders the development of methylation studies in every aspect. It is urgent to establish standardized protocols from sample storage, ctDNA/RNA extraction to methylation analysis. Translating circulating epigenetic biomarkers from clinical study to clinical routine for lung cancer is expected.

## Abbreviations

AIM1: Absent in melanoma 1
APC: Adenomatous polyposis coli
BRMS1: Breast cancer metastasis suppressor-1
caCpGs: Lung cancer-related CpGs
CDH1: Cadherin 1
CDH13: Cadherin 13
CDKN2A: Cyclin dependent kinase inhibitor 2A
cfDNA: Circulating cell-free DNA
CGIs: CpG islands
CHFR: Checkpoint with forkhead and ring finger domains
COBRA: Combined Bisulfite Restriction Analysis
csbDNA: Cell-surface-bound circulating DNA
CSF3R: Colony stimulating factor 3 receptor
CTCs: Circulating tumor cells
ctDNA: Circulating tumor DNA
CXCL14: C-X-C motif chemokine ligand 14
DAPK: Death-associated protein kinase
DCC: DCC netrin 1 receptor
DCLK1: Doublecortin like kinase 1
DLEC1: Deleted in lung and esophageal cancer 1
DMH: Differential methylation hybridization
EBC: Exhaled breath condensate
ERCC1: ERCC excision repair 1
FHIT: Fragile histidine triad
F-MSP: Fluorescent methylation-specific PCR
GATA5: GATA binding protein 5
GSTP1: Glutathione S-transferase P1
HELP: HpaII tiny fragment enrichment by ligation-mediated PCR
HM450K: HumanMethylation450K BeadChip Assay
HOXD10: Homeobox D10
IEAA: Intrinsic epigenetic age acceleration
Jph3: Junctophilin 3
KLK1: Kallikrein 1
LC: Lung cancer
LDCT: Low-dose computed tomography
MBD: MethylCpG Binding Domain MBD2 proteins
MCAM: MCA with microarray hybridization
MeDIP: Methylated DNA immunoprecipitation
MGMT: O6 - methylguanine-DNA-methyltransferase
MIRA: Methylated CpG island recovery assay
MS-MLPA: Methylation-specific multiplex ligation-dependent probe amplification
MSP: Methylation-specific PCR
MSREs: Methylation-Sensitive Restriction Enzymes
NA: Not available
NGS: Next-generation sequencing
OS: Overall survival
p16: Cyclin-dependent kinase inhibitor 2A
PAX5a: Paired box 5a
PAX5b: Paired box 5b
PAX9: Paired box 9
PCDH20: Protocadherin 20
PCDHGB6: Protocadherin gamma subfamily B, 6
PFS: Progression free survival
PTGER4: Prostaglandin E receptor 4
PTPRN2: Protein tyrosine phosphatase receptor type N2
qMSP: Quantitative methylation-specific polymerase chain reaction
qPCR: Quantitative PCR
RARβ2: Retinoic acid receptor B2
RASSF1A: Ras association domain family 1 isoform A
RTEL1: Regulator of telomere elongation helicase 1
RTL: Relative telomere length
SCLC: Small cell lung cancer
SEPT9: Septin9
SFN: Stratifin
SHOX2: Short stature homebox 2
smoCpGs: Smoking-associated CpGs
SOX17: (sex determining region Y)-box 17
STAG3: Stromal antigen 3
SULF2: Sulfatase 2
TSS: Transcription start site
WGSBS: Whole-genome shotgun bisulfite sequencing
